# Flatfoot Diagnosis by a Unique Bimodal Distribution of Footprint Index in Children

**DOI:** 10.1371/journal.pone.0115808

**Published:** 2014-12-31

**Authors:** Chia-Hsieh Chang, Yu-Chen Chen, Wen-Tien Yang, Pei-Chi Ho, Ai-Wen Hwang, Chien-Hung Chen, Jia-Hao Chang, Liang-Wey Chang

**Affiliations:** 1 Institute of Biomedical Engineering, College of Medicine and College of Engineering, National Taiwan University, Taipei, Taiwan; 2 Department of Pediatric Orthopedics, Chang Gung Memorial Hospital, Chang Gung University, Taoyuan, Taiwan; 3 Taipei Chengshih University of Science and Technology, Taipei, Taiwan; 4 Graduate Institute of Early Intervention, College of Medicine, Chang Gung University, Taoyuan, Taiwan; 5 Department of Physical Education, National Taiwan Normal University, Taipei, Taiwan; Medical University of South Carolina, United States of America

## Abstract

**Background:**

More than 1000 scientific papers have been devoted to flatfoot issue. However, a bimodal distribution of flatfoot indices in school-aged children has never been discovered. The purposes of this study were to establish a new classification of flatfoot by characteristic in frequency distribution of footprint index and to endue the classification with discrepancy in physical fitness.

**Methods/Principal Findings:**

In a longitudinal survey of physical fitness and body structure, weight bearing footprints and 3 physical fitness related tests were measured in 1228 school-aged children. Frequency distribution of initial data was tested by Kolmogorov-Smirnov test for normality and a unique bimodal distribution of footprint index was identified. The frequency distribution of footprint index manifests two distinct modes, flatfoot and non-flatfoot, by deconvolution and bootstrapping procedures. A constant intersection value of 1.0 in Staheli's arch index and 0.6 in Chippaux-Smirak index could distinguish the two modes of children, and the value was constant in different age, sex, and weight status. The performance of the one leg balance was inferior in flatfoot girls (median, 4.0 seconds in flatfoot girls vs. 4.3 seconds in non-flatfoot girls, p = 0.04, 95% CI 0.404–0.484).

**Discussion:**

The natural bimodality lends itself to a flatfoot classification. Bimodality suggests development of the child's foot arch would be a leap from one state to another, rather than a continuous growth as body height and weight. The underlying dynamics of the human foot arch and motor development will trigger research prospects.

## Introduction

The human foot arch is a complex structure offering elasticity for shock absorption as well as stability for transmitting muscle force while walking. Flatfoot is a depression of the medial longitudinal foot arch. Insufficiency in foot arch function in adults may increase the risk of overuse injury [1]–[4]. The overuse injury in adults with flatfeet is one of the causes of posterior tibial tendon dysfunction and chronic symptoms [5], [6].

Flatfoot in children is often a dynamic and restorable depression of the foot arch during weight bearing, also known as flexible flatfoot. Flexible flatfoot has been regarded as a physiological deviation rather than a disorder [7]–[10], and the foot arch develops along with body growth in children [9]. However some of the children with flatfeet might not develop a good foot arch at skeletal maturity, and inferior physical fitness was reported in children with flatfeet [2], [11]–[12].

Flatness of the foot arch has been a controversial issue in its diagnosis and management. The weight-bearing footprint was the most common measurement, particularly for large-scale studies because it is simple and intuitional. However, several classification systems of footprint have been produced to define flatfoot [2]–[3], [7], [13]–[16] and the diagnosis could be different depending on which classification was used [17]–[18]. Lack of a universal diagnosis of flatfoot has made any management to elevate foot arch or to enhance arch development in children controversial. Before the controversy can be resolved, an objective classification of foot flatness is required.

Here we report a bimodal distribution of the footprint measurements in elementary school children, while their height, weight, and foot length were all in unimodal distribution. The purposes of this study were to establish a new classification of foot flatness by a unique characteristic in frequency distribution of footprint data and to endue the classification with physiological significance.

## Methods

In a field survey of physical fitness of the students in first and second grades in 3 suburban elementary schools in Taipei, Taiwan, the research team collected weight bearing footprints. Age, sex, body height, body weight, and body mass index (BMI) were recorded. Children with major medical diseases such as diabetes mellitus, diseases in heart, liver and kidney, cancer, and cerebral palsy that could affect physical fitness were not included. Children with musculoskeletal disorders such as clubfoot, limb deficiency, and leg length discrepancy that could affect measurements of footprint and physical fitness were excluded.

Three related tests of physical fitness, including the 20-meter dash, the standing long jump, and the one leg balance were measured. The 20-meter dash was a measurement of muscle strength and speed. Each subject dashed for 20 meters from standing position with time recorded to the tenth of a second. The standing long jump was a measure of explosiveness of the leg muscles. Each subject stood behind a marked line keeping both feet at shoulder width apart. Subjects took off for a jump using both feet simultaneously. The distance from the starting line to the nearer landing foot was recorded by millimeters. The one leg balance was a measure of mixed abilities of visual, vestibular and musculoskeletal control. Each subject with eyes open stood barefooted by one foot on a 30-cm long wood block (3 cm×3 cm×30 cm). The longitudinal axis of the foot was aligned with the block length. Arm movement for balance was allowed. The time from taking the other foot off to touching the ground again was recorded to the tenth of a second.

### Footprint recording

Each subject was asked to put both feet on two Harris and Beath footprint mats [15]–[16] while sitting on a chair. The subject then stood up to a standing position with even weight on both feet, and returned to sitting position to complete the footprint recording. Footprint data were rejected when apparent overshoot or imbalance had occurred on standing up or significant foot movement had occurred during recording. Footprint data from the right foot was used to represent a subject.

### Footprint measurements

**Figure 1:**
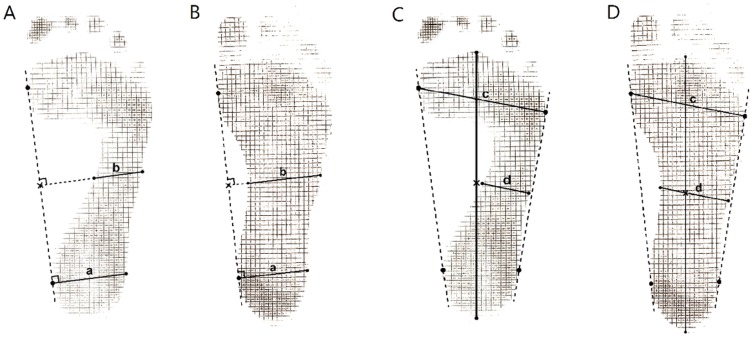
Measure of SAI = b/a in non-flatfoot (A) and flatfoot (B); measure of CSI = d/c in non-flatfoot (C) and flatfoot (D).

Staheli's arch index (SAI) [10], [20] and Chippaux-Smirak index (CSI) [14], [17] were employed to measure flatness of the footprint. Three basic lines, the medial tangential line, the lateral tangential line, and the longitudinal axis were drawn on footprints first. The medial tangential line was marked by the most medial points at the metatarsal and the heel. The lateral tangential line was defined by the most lateral points at the metatarsal and the heel. The longitudinal axis of the foot was a line connecting the 2nd metatarsal head and the heel tip. A heel line was drawn from the most medial point of the heel, perpendicular to the medial tangential line, to the lateral border of heel. The heel width (a) was readily defined by the heel section on the heel line. A midfoot line was drawn from the midpoint of the medial tangential line and parallel to the heel line. The midfoot width (b) was defined as the length of the foot section on the midfoot line. The metatarsal width was measured from the most lateral point to the most medial point of the forefoot (c). SAI is the ratio of midfoot width to heel width (b/a). CSI is the ratio of midfoot width to metatarsal width (b/c) (Fig. 1). The midfoot level was not taken by the narrowest part of the midfoot to avoid the subjectivity. The foot arch index measurement had excellent inter-rater reliability (ICC: 0.95 in SAI and ICC: 0.98 in CSI) and test-retest reliability (ICC: 0.96 in SAI and ICC: 0.97 in CSI).

### Statistical analysis

The frequency distribution of subjects' body height, weight, 3 physical fitness tests, foot length, foot width, and arch index was described by parameters such as mean, median, and skewness. For distribution curves with bimodal appearance and a marked deviation from the theoretical normal curve, deconvolution was made to produce 2 best-fitted normally distributed curves with the EM algorithm [21] with the R statistical package (R Version 2.7.2, The R Foundation for Statistical Computing, 2008). The deconvolution was ensured by bootstrapping with 1000 replications of the original data to obtain the mean values and standard errors of two normal distribution curves. The bootstrapping also yielded an estimation of the intersection point between the two component normal distribution curves. The deconvolution and bootstrapping were also performed in subgroups of children by their age, gender and weight status to test the consistency of bimodality and intersectional value in each characteristic subgroup. The value of intersection point could serve as diagnostic criteria of flatfoot.

By the intersectional value, the first grade students were classified into flatfoot and non-flatfoot based on their right foot data. Since flexible flatfoot was associated with age, gender, and obesity [17], [20], [22]–[26], we examined these associations to validate the newly emerged diagnostic criteria of flatfoot. The association between gender and flatfoot was analyzed by chi-square test. Age, body mass index (BMI), and fitness tests were first determined on the normality of distributions by Kolmogorov-Smirnov test [27]. Then independent t test was used for group comparison when the normality assumption was satisfied; otherwise, Mann-Whitney U test was applied. All α levels are set at 0.05.

### Ethics Statement

The protocol for this survey was reviewed and approved by the ethic committee (Institutional Review Board) of the Chang Gung Memorial Hospital in Taoyuan, Taiwan. The observation study was registered in Chinese Clinical Trial Registry (ChiCTR-OCS-14004300).

## Results

**Figure 2:**
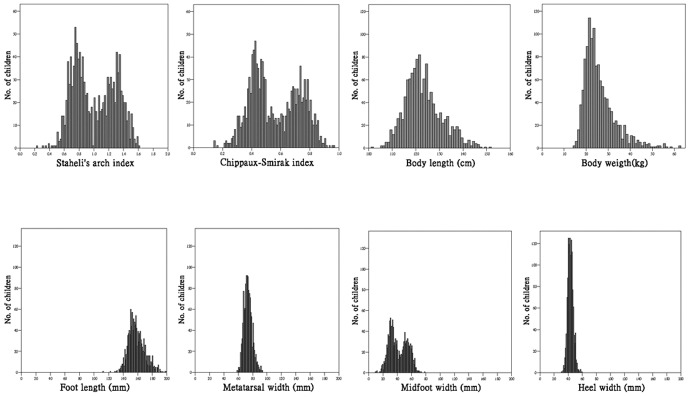
Frequency distributions of body measures in 1228 children.

We collected data of physical fitness and weight-bearing footprints from 1228 elementary school students. There were 638 boys and 590 girls. Their age ranged from 6.1 to 9.9 years (mean age 7.3, SD 1.1). Frequency distributions of body weight, BMI, and 3 physical fitness tests were all in unimodal distribution and positively skewed. Frequency distributions of the SAI and CSI values in the 1228 children conformed to a bimodal distribution pattern. The bimodal distribution of footprint index was resulted from bimodality in midfoot width. The width of the metatarsal and the width of the heel were unimodal, as were their body height and weight (Fig. 2).

**Figure 3:**
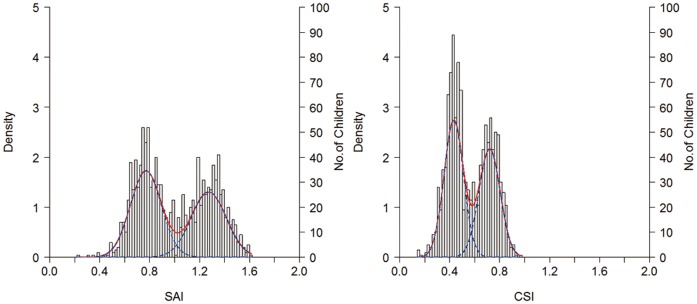
Two normally distributed curves (blue lines) obtained from deconvolution process are combined to represent a theoretical frequency curve (red line).

The bimodal distribution curve indicates two embedded modes from two distinct populations. The right mode portrays the distribution of the children with greater footprint index, the flatfoot mode. The left mode portrays the children with smaller footprint index, the non-flatfoot mode. In SAI, the non-flatfoot mode had a mean value of 0.77 (SD = 0.13), and the flatfoot mode had a mean value of 1.28 (SD = 0.14). In CSI, the mean of non-flatfoot mode was 0.43 (SD = 0.08) and the mean of flatfoot mode was 0.73 (SD = 0.08). The intersection of the two modes was found at the value of 1.02 (95% CI 0.99–1.04) in SAI and 0.59 (95% CI 0.58–0.61) in CSI. (Fig. 3).

**Figure 4:**
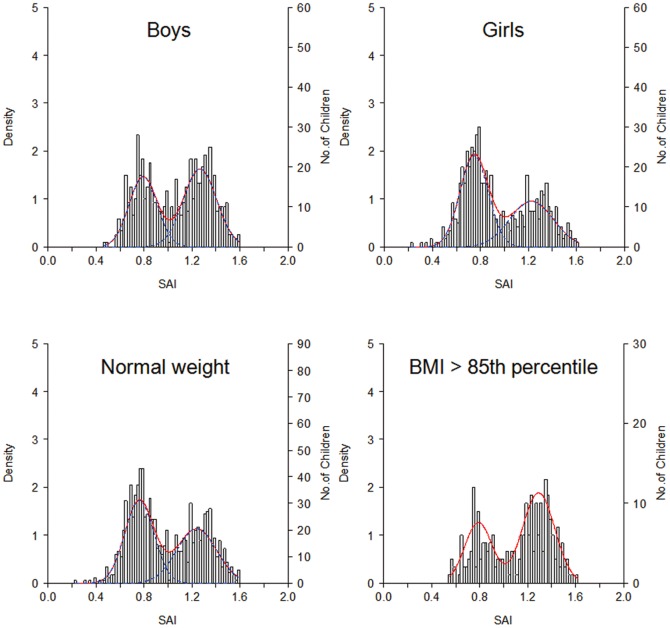
Bimodality of SAI in different genders and weight status. The intersection points are constantly around 1.0.

Bimodal distribution of footprint index also existed in boys, girls, overweight children (BMI≧85th percentile), and subgroups of age 6, 7, 8, 9 years. The bimodality is a universal phenomenon in the immature foot structure and prevails regardless of sex, age, and obesity that were well-known factors associated with flatfoot (Fig. 4).

**Figure 5:**
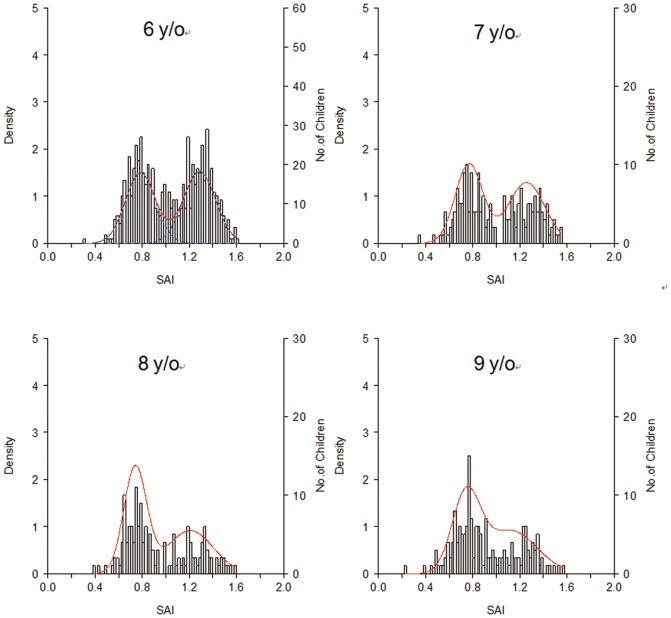
Distribution of SAI data in different ages. Children of age of 6 years (n = 695), 7 years (n = 202), 8 years (n = 159) and 9 years (n = 172).

Bootstrapping revealed the intersectional value remained constant as SAI 1.0 and CSI 0.6 in boys, girls, and normal weight children in 6-year-old children. Bootstrapping procedures were not applied to the age bands of 7 (n = 202), 8 (n = 159), and 9 (n = 72) years due to small sample size. Nevertheless SAI 1.0 was located exactly at the trough of each theoretical frequency curve, indicating a consistent intersection value existed to differentiate flatfoot mode and non-flatfoot mode (Fig. 5). Using SAI 1.0 to divide the two modes, the percentage of flatfoot children is 51.4%, 46.7%, 36.9%, and 32.4% at the ages of 6, 7, 8, and 9 years, respectively. It apparently shows a natural maturation course of the foot arch development in school-aged children.

Using SAI 1.0 to classify foot flatness in the 1228 children with age from 6.1 to 9.9 years, there were 556 children in flatfoot mode and 662 children in non-flatfoot mode. Flatfoot children had older age (7.5 years vs. 7.2 years in non-flatfoot children, p<0.001, t test), greater BMI (16.3 vs. 15.8 in non-flatfoot children, p<0.001, t test), and greater percentage of boys (62.6% vs. 43.8% in non-flatfoot children, p<0.001, chi-square test). The results were comparable to previous reports [18], [20], [22]–[24].

Table 1 listed the performance of 3 physical fitness tests in flatfoot and non-flatfoot children after stratifying age and sex. The duration of staying on the wood rod in the one leg balance test was significantly longer in non-flatfoot girls (median, 4.0 seconds in flatfoot girls vs. 4.3 seconds in non-flatfoot girls, Mann-Whitney U test, p = 0.04, 95% CI 0.404–0.484) (Table 1).

**Table 1:** Medians and inter-quarter ranges of physical fitness performance were compared between flatfoot and non-flatfoot children after stratifying age and sex.

First grade students (n = 853)	Flatfoot (n = 446)	Non-flatfoot (n = 407)	p value
Age (years)	6.68	6.70	0.3^a^
Boys (n = 440)			
20-meter splint (sec)	5.1 (4.7∼5.4)	5.1 (4.7∼5.3)	0.12^b^
Standing long jump (cm)	110.0 (100∼120)	110.0 (100∼120)	0.30^b^
One leg balance (sec)	3.9 (2.7∼6.3)	3.9 (2.8∼6.9)	0.27^b^
Girls (n = 413)			
20-meter splint (sec)	5.3 (4.9∼5.7)	5.3 (4.9∼5.6)	0.20^b^
Standing long jump (cm)	100.0 (90∼110)	100.0 (95∼110)	0.23^b^
One leg balance (sec)	4.3 (2.9∼8.1)	4.0 (2.8∼6.2)	0.04^b^

a t-test.

b Mann-Whitney *U* Test.

## Discussion

Flatness of the foot is one of the features of human body, as height, weight, and girth that are commonly measured as an index of health condition in children. It has been a clinical controversy for a long time concerning what degree of flatness constitutes a flat foot. Because of a lack of a clear clinical diagnostic gold standard, it is debatable to classify foot flatness by a continuous variable of footprint index. Besides, many studies classified flatness of the foot and analyzed footprint data based on normal distribution assumption [7], [10], [17]–[19], [23], [28]. The present study reveals a unique nature of bimodal distribution in footprint indices. The findings open up a new perspective that healthcare professionals and biomechanicians could re-define the flatfoot issue and move forth to unveil the truth behind the foot arch-index bimodality.

Questions might be asked on why the atypical distribution was not discovered before. We collected footprints from large number of children whose ages were limited in 6 to 7 years. It is a stage in the middle of the foot arch development and shows a remarkable 1∶1 ratio of flatfoot mode to non-flatfoot mode. When children grow older, as the distribution of 9-year-old children shown in Fig. 5, the flatfoot mode becomes much smaller and the distribution could be neglected as unimodal. One can speculate that if our subjects were preschool children, the flatfoot mode would have been dominant.

The flatfoot mode fades as the children grow older indicating it is a physiological development of the foot arch. However, the strong bimodality suggests the foot arch development should not be regarded as a continuous process, akin to increases in body height and weight. We speculate a leap in the foot-structural development just as infants and toddlers developing a new motor skill. These developments might occur suddenly and each child has his or her time of onset. Further longitudinal cohort studies with series of footprint recordings are required to prove the above speculation.

By the new classification of flatfoot mode, this study revealed that flatfoot girls had inferior performance in one leg balance. Different from the one leg balance tested by standing on the ground in Tudor's study [19], subjects in this study were tested by standing on a long square rod. The present test puts more stress on the coronal stability and muscle control of the ankle. Excessive eversion of the ankle is a common deformity in flatfoot [2]–[4]. The insufficiency in ankle joint stability in flatfoot girls might be one of the reasons of inferior performance in one leg balance.

There are limitations of this study. First, right foot data was used to represent a subject in this study. However, some children had one flatfoot and one non-flatfoot. Children with asymmetrical foot arch development might be in a transitional status that requires further study to prove. Second, handedness might affect footprint recording because children tend to put more weight on their dominant legs during standing up. In the fact that flatfoot mode transited from 51% to 32% in children with age from 6 to 9 years, bimodality would be a characteristic in body development rather than a result of handedness. Third, this cross-sectional survey revealed the association between flatfoot and ankle motor control. The causal pathway requires longitudinal survey or interventional study to define.

## Conclusions

The study revealed how well the natural bimodality lends itself to the classification of footprint in children. The bimodality suggests that the development of human foot structure is not a continuous process as gaining height and weight, but rather a leap from one state to another. The underlying dynamics of the foot arch development and the associated motor control of the ankle will trigger exciting research prospects.
